# Provider perception of presentations with nonspecific back pain in the emergency department and primary care practices: a semi-structured interview study

**DOI:** 10.1186/s12245-024-00694-2

**Published:** 2024-09-11

**Authors:** Leo Benning, Nora Köhne, Hans-Jörg Busch, Felix Patricius Hans

**Affiliations:** 1 University Emergency Center, Medical Center – University of Freiburg, Sir-Hans-A.-Krebs-Straße, 79106 Freiburg, Germany; 2 Emergency Department Campus Virchow-Klinikum Internal Medicine, Charité – Universitätsmedizin Berlin, corporate member of Freie Universität Berlin and Humboldt-University Berlin, Augustenburger Platz 1, 13353 Berlin, Germany

**Keywords:** Health resources, Triage, Health personnel, Emergency Medicine, Interview, Low back Pain, Physician-patient relations

## Abstract

**Background:**

Increasing numbers of patients treated in the emergency departments pose challenges to delivering timely and high-quality care. Particularly, the presentation of patients with low-urgency complaints consumes resources needed for patients with higher urgency. In this context, patients with non-specific back pain (NSBP) often present to emergency departments instead of primary care providers. While patient perspectives are well understood, this study aims to add a provider perspective on the diagnostic and therapeutic approach for NSBP in emergency and primary care settings.

**Methods:**

In a qualitative content analysis, we interviewed seven Emergency Physicians (EP) and nine General Practitioners (GP) using a semi-structured interview to assess the diagnostic and therapeutic approach to patients with NSBP in emergency departments and primary care practices. A hypothetical case of NSBP was presented to the interviewees, followed by questions on their diagnostic and therapeutic approaches. Recruitment was stopped after reaching saturation of the qualitative content analysis. Reporting this work follows the consolidated criteria for reporting qualitative research (COREQ) checklist.

**Results:**

EPs applied two different strategies for the workup of NSBP. A subset pursued a guideline-compliant diagnostic approach, ruling out critical conditions and managing pain without extensive diagnostics. Another group of EPs applied a more extensive approach, including extensive diagnostic resources and specialist consultations. GPs emphasized physical examinations and stepwise treatment, including scheduled follow-ups and a better knowledge of the patient history to guide diagnostics and therapy. Both groups attribute ED visits for NSBP to patient related and healthcare system related factors: lack of understanding of healthcare structures, convenience, demand for immediate diagnostics, and fear of serious conditions. Furthermore, both groups reported an ill-suited healthcare infrastructure with insufficiently available primary care services as a contributing factor.

**Conclusions:**

The study highlights a need for improving guideline adherence in younger EPs and better patient education on the healthcare infrastructure. Furthermore, improving access and availability of primary care services could reduce ED visits of patients with NSBP.

**Trial registration:**

No trial registration needed.

**Supplementary Information:**

The online version contains supplementary material available at 10.1186/s12245-024-00694-2.

## Background

Emergency departments (ED) face increasing patient volumes, which poses challenges to delivering timely and effective care [1]. While EDs are equipped to serve patients with severe conditions, low-urgency visits to the ED bear risks for patients, the ED staff and the health care system, summarized in the concept of ED crowding [2, 3]. During episodes of ED crowding, patients are at risk since the allocation of available and required resources is constrained. Thus, low-urgent presentations tie up capacities required for treating acutely ill patients [4], while the risk for medical errors rises [5, 6]. Additionally, high patient volumes extend overall waiting times and delay the admission of patients who need inpatient care [7]. The latter also affects the morbidity and mortality of these patients [8, 9]. Likewise, long waiting times increase the risk for patients to leave without being seen by a qualified medical professional, which does not address the medical need of these patients [10] and puts them at a higher risk for adverse consequences [11]. Simultaneously, staff working in EDs experience high stress during crowding, which leads to low workplace satisfaction, an increased prevalence of burnout and the intention to leave (ITL) [12]. Lastly, crowding burdens the healthcare system, as the treatment of low-urgent patients in the ED incurs costs that are unnecessary to provide adequate care for these patients [13]. Efforts to reduce the influx of low-urgency patients have included explicit referrals to primary care practices and urgent care practices [14], the latter being an approach that is currently emphasized in the Germany healthcare system through novel legislation [15]. Internationally, the option to have physiotherapists available in EDs has furthermore demonstrated promising results [16].

Although patients with back pain presenting to an ED have a higher prevalence of serious underlying causes or might require more extensive diagnostic measures for other reasons (e.g. comorbidities, inability to express their complaints), prior work in this field identified non-specific back pain (NSBP) as a particularly frequent condition among low-urgency patients [17–19]. While patient perspectives and healthcare system-related factors driving non-urgent ED visits have been explored extensively [20–25], this work adds a provider perspective on how patients with NSBP are perceived, how the workup of patients with NSBP is conducted, and what similarities and differences exist between the ED and the primary care setting. This work therefore presents a semi-structured interview of General Practitioners (GP) and ED physicians (EP) in Germany who work within the catchment area of one of the country’s largest tertiary care emergency centers, or in the emergency center, respectively.

## Methods

To analyze the diagnostic and therapeutic workup of patients presenting with NSBP and the assumed causes for presentations to the ED, we conducted a semi-structured interview with physicians working as EPs or as GPs. Participating EPs were recruited from a large, tertiary level emergency center, while GPs were recruited from the catchment area of the respective center. The recruitment material was posted in a weekly center-wide newsletter and sent out to GPs within the catchment area of the emergency center via email. Participants were purposefully sampled in a non-probabilistic manner to represent the two groups of acute care providers. No additional criteria had to be met.

### Development of interview

The semi-structured interview was designed for qualitative content analysis and included a case presentation with five open questions that covered the diagnostic and therapeutic approach, further recommendations and assumptions for why patients present to the ED (Supplementary Table 1). The interviewer introduced an NSBP case with a medical history suggesting a benign condition (i.e. not providing any evidence of “red flags” [26]). Participants were allowed to ask clarifying questions before discussing the interview questions. The interview was pilot-tested on two individuals, each representing one of the above groups of physicians, and underwent minor revisions to increase the precision and comprehensibility.

### Ethics approval and consenting

Interview participants were recruited through publicly posted information material. Upon indicating interest, eligible participants were provided with information on the purpose, content and use of the interview. All interview participants gave their informed consent to their participation in the interviews, the recording, analysis, and publication of the findings. To facilitate a low-barrier and remote participation in the interviews, consent was given verbally and recorded prior to conducting the interview. The study was submitted to the ethics committee of the Albert-Ludwigs-Universität Freiburg. An ethics approval was waived by the ethics committee under the reference *24-1231-Anfrage*.

### Data collection, analysis, and reporting

The interviews were conducted remotely between May 12 and June 6 2023 and recorded as audio files, either by using a video communication software (Zoom Video Communications, Inc., San José, USA), or by telephone. For each interview, the interviewer and the respective interviewee met without other persons present. Each interviewee was interviewed once. Transcriptions of the audio files were prepared by a writing agency (amanu GmbH, Stuttgart, Germany), anonymized and sent to the authors for proofreading. The transcripts were screened for aspects (codes) assigned by the interviewees using qualitative data analysis software (MaxQDA 2022, Verbi Software, Berlin, Germany). In an incremental, iterative process, aspects were collected in a codebook under constant monitoring of data saturation by assessing the number of new aspects per interview [27]. Preparation of this manuscript followed the COREQ checklist [28], which is provided as Supplementary Table 2.

## Results

### Baseline characteristics

Sixteen interviews were conducted, seven with emergency physicians from the same emergency center and nine with general practitioners from primary care practices in the catchment area of the center. The EP group consisted of three female doctors (42.86%) and four male doctors. The interviewees’ mean age was 37 years (31 to 48). The mean work experience of EPs was nine years (3.5 to 20), and the mean interview duration was 09:36 min (7:35 − 12:29). The GP group comprised four female (44.44%) and five male interviewees. The mean age of this group was 56 years (37 to 74), and the mean work experience was 26 years (10 to 40). The mean interview duration in the GP group was 11:24 min (07:40 to 16:55). The baseline characteristics of the participants are given in Table 1.

**Table 1:** Baseline characteristics of the *N* = 16 interviewees

Category	Total	ED	GP Practice
Number of Interviews (*n*, %)	16 100%	7 43.75%	9 56.25%
Female gender (*n*, %)	7 43.75%	3 42.86%	4 44.44%
Age in years (mean, min., max.)	48 31–74	37 31–48	56 37–74
Work experience in years (mean, min., max.)	18.44 3.5–40	9 3.5–20	26 10–40
Interview duration in minutes (mean, min., max)	10:36 7:35 − 16:55	09:36 7:35 − 12:29	11:24 7:40 − 16:55

### Emergency physicians’ perspective

#### Diagnostic and therapeutic workup

When a patient with NSBP presents to the ED, two different diagnostic approaches of EPs were identified. *“Typically*, *you would check for neurological deficits […]. The only thing we do is give them analgesics and do not even do any imaging and send them home again.”(I3)*.

This first approach was based on a physical examination and tests for neurological deficits and did not include diagnostic imaging or blood tests because “*I do not need a lab for that*.”(I9).

“*So in the emergency room*, *I would*, *of course*, *do a physical examination and a lab anyway. And I would also do an imaging scan*, *so an X-ray or something. And then*, *of course*, *as a consultant*, *I also have a short route to the trauma surgeons so that they can have a look*.”(I7). The second approach was characterized by a more extensive use of diagnostics and resources in the ED to assess the patients’ symptoms beyond the medical history and physical examination through further diagnostic measures. These measures included blood tets to look for signs of infection, or performing diagnostic imaging (e.g. X-ray, CT Scan). A consultation with the on-call trauma surgeons was suggested.

EPs’ therapeutic approach comprised two main aspects. One aspect was the analgesic therapy, and the second aspect was the apparent need for a psychosocial intervention as “…*there are also patients who*, *let us say*, *objectively*, *do not have so much of a problem*, *or the pain is perhaps not that severe*, *but they suffer a lot. I would not treat them with intravenous pain medication*, *but rather have a bit of a conversation and try to see what other stress factors they might have*.”(I6). This conversation provided stress relief for patients who “…*ultimately require counselling…*” (I9).

The main recommendation for further treatment from the EPs was a follow-up visit with the patient’s GP: “…*the patient should actually see their GP on the next working day*, *would be my recommendation so that the GP sees the symptoms with which they presented to the emergency department*.” (I8). Furthermore, EPs provided education about beneficial additional treatment aspects like physiotherapy and keeping up moderate movement and how diagnostic imaging should only be considered if there is a lack of improvement over time. Diagnostic Imaging here referred to performing an X-ray or, if an X-ray was performed in the ED, a CT Scan or MRI.

#### Resource utilization

As reported by the interviewees, the main difference in care was that a wide range of resources were available in the ED. The fact that blood tests, X-rays, and even CT scans can be performed within hours is an advantage of care in the ED. Additionally, the availability of other specialists led to an extensive patient assessment. The availability of resources alone is reported to increase the chance of utilization in the ED (“*If I can draw on unlimited resources*, *then I will just do it*.”(I12)). In a sense, the care and resource use depended on the individual EP (“*I think it just depends on which person is doing the job at the time*, *whether they want to do it well or not*.” (I8)). One ED doctor stated: “*In the emergency department*, *I see the patient for the first time and do not have time [for] a detailed medical history*, *so I do first-time diagnostics*.”(I10).

Other reported factors were patient-specific circumstances like severe pain and high emotional distress. To “…*take them out of the stressful situation for a short time*” (I3), the doctor decided to administer intravenous analgesia.

EPs also reported higher patient satisfaction when an X-ray was ordered, or diagnostic measures were used to bridge waiting times. Yet, EPs also considered medico-legal aspects when utilizing diagnostic resources and aimed to avoid missing a serious condition they could have diagnosed, given that they had access to the required diagnostic means. In this light, extensive diagnostics were perceived as useful “…*to send him on a safe path to outpatient care*” (I8). It seemed, however, clear for EPs that higher resource consumption did not necessarily lead to better care. One EP sarcastically stated: “*Everyone needs a [blood] lab [test] and an X-ray; otherwise*, *they are not being treated properly; otherwise*, *we do not even know what they have*.” (I12).

#### Assumed reasons for presentations to ED

Emergency physicians assumed that infrastructural deficits in the primary care sector drive patients with NSBP into EDs. To access nearby primary care services, “…*you have to spend ages researching it*” (I9), one EP stated, referring to where to find the next GP practice, what opening hours it has, and how an off-hour urgent care practice, typically covering the time between 5 pm and midnight for primary care services, can be found. Also, EPs perceived that long waiting intervals for appointments with GPs or other specialists in the primary care sector were among possible healthcare-related reasons for patients with NSBP to divert into EDs. Furthermore, EPs assumed a general dissatisfaction of patients with primary care.

Among patient-related factors, convenience was considered important, as the ED presentation was perceived as “*uncomplicated*” (I5) and “…*[the] lowest threshold for going somewhere is the emergency room*.”(I5). Additionally, the around-the-clock availability, the wish for a timely resolution of the symptoms and an increased demand for certain diagnostics may drive the primary presentation in the ED. This aspect was reflected by a GP quoting a patient: “*Now I’m going to the university hospital’s emergency department because they have an MRI [scanner] there*.”(I9).

A lack of knowledge of the healthcare system was thought to be another reason, as EPs assumed that not being registered with a GP or being reluctant to do so might be another driver for ED attendance. Yet, one EP also highlighted that some patients have a very good understanding of the healthcare system and its challenges to provide timely primary care. This was consistent with the statement, “[…] *there is certainly the other smaller group who know what the system is like and do not want to wait for a GP or look for one*.”(I8).

### General practitioner’s perspective

#### Diagnostic and therapeutic workup

The patient with NSBP presenting to General Practice was examined physically and tested for neurological deficit. “*I would first clarify whether there are any neurological complaints*, *whether there is a radiation of pain. If there is none of this*, *but only localized back pain*, *I would probably first treat it with some kind of anti-inflammatory treatment and accompany it with physiotherapy to see if it gets better*.” (I15) one GP stated. This approach was similar to one of the EPs approaches, which eliminated red flags, initiated treatment and then sent the patient home.

Some GPs ordered an X-ray or performed other diagnostic measures e.g. ultrasound or urinalysis, depending on, for example, age at the first time occurrence of the symptoms (“*If* […] *patients are between 50 and 60 and have never had an X-ray*, *then you could consider a lumbar spine X-ray if [the pain] is in the lumbar spine area*.” (I16)).

The therapeutic approach pursued by GPs consists of symptomatic therapy with analgesics. They often provided counselling in “…*a somewhat calmer atmosphere*, *to educate the patient*, *to discuss things with them*, *to discuss strategies with them on how they can deal with* [NSBP]” (I15).

Regarding recommendations for further treatment, GPs reported a variety of possibilities: Pain medication, physiotherapy, and advice to stay active, avoid non-ergonomic movements and use local heat applications. GPs regularly recommended a follow-up visit in a few days to reevaluate the situation. An important aspect for the GPs was “*In any case*, *education is essential. Talk to the people*.”(I10).

#### Resource utilization

The setting in General Practice differed from the setting in the ED (“*In fact*, *the practitioner knows the previous illnesses*, *knows the course of the disease and can assess the person’s character well. And he knows exactly when […] something is wrong with him*.”(I4)). The patient history is known, and a doctor-patient relationship has already been established. Ideally, the GPs could allocate more time to each patient, too.

The strength of the care in general practice, from the perception of the GPs interviewed, lies in the possibility of performing gradual diagnostics and therapy over weeks or months. This is reflected by the statement: “*The further procedure then consists of the patient possibly becoming better after acute treatment*, *in which case he will no longer turn up. Or […] symptoms persist*, *then he will present again and then*, *in principle*, *perhaps an extended diagnosis with X-ray*, *MRI*, *CT*, *et cetera or differential diagnostics in the direction of radiating symptoms et cetera will take place. And then*, *in principle*, *further treatment usually involves physiotherapy and further pain therapy. If that does not work*, *the patient is scheduled for outpatient or inpatient rehabilitation. The GPs can do this themselves or*, *if*, *let us say*, *the necessary expertise is not available in general practice*, *then a referral is made to an orthopedic practice*.” (I11).

GPs reported a network of ambulant colleagues to whom they could refer patients, get appointments and ask for advice because “*You are quite well-connected*.”(I10). Furthermore, as resources like diagnostic imaging or blood tests were perceived to not be readily available, the GP relied on “…*my clinical skills and my experience*, *which is of course usually sufficient for me personally*.” (I10). From the GPs’ perspective, the care for NSBP belonged to primary care. One GP stated: “[Patients with NSBP] *burden colleagues in the emergency department. That should not be the case. They should all come to us*, *in my opinion*.”(I14).

#### Assumed reasons for presentation to the ED

Consistent with the suspected reasons by EPs, GPs assumed patient-related, healthcare-related and administrative aspects to be involved in the reasons for ED presentations of patients with NSBP.

A major assumed reason was the insufficient knowledge of emergency care structures and the health care system. The GPs perceived that patients presented to the ED because they “…*do not know enough about our care system*, *that many things do not necessarily need to be treated in the emergency room*” (I15). At the same time, the 24/7 availability of EDs, and insufficient public communication about entry points into primary care possibly made it harder for patients to navigate the healthcare system in a state of subjective urgency.

The GPs suspected convenience aspects, as an interviewee described ED presentations as the “‘…*Amazon shopping in medicine - they want it 24/7”* (I12). Increased demand for imaging diagnostics, prompt administration of pain medication and timely resolution of complaints were equally seen as main drivers. Also, the ED might be attributed as an adequate choice to access the healthcare infrastructure primarily. One interviewee described these patients’ perception as “…*the university hospital is my family doctor*.” (12). Choosing the ED as a primary care provider was “[…] *completely inadequate*, *yes*, *but that is how it is done*.” (I12).

From the GPs’ perception, insufficient infrastructure and service levels in the ambulatory sector in general and primary care in particular were possible reasons for presentations to EDs. Hence, patients divert to EDs due to long waiting times, insufficient access to appointments with their GP or general dissatisfaction with their ambulant care provider.

Lastly, uncertainties and worries were stated as possible reasons for ED presentations. Patients with NSBP might be “…*the classic case […] who is worried*” (I15) that they have a serious condition and need a fast resolution of their symptoms, as described aptly with “*It hurts me*, *I want to know now*.” (I1).

## Discussion

Our study employed semi-structured interviews with EPs and GPs to evaluate the utilization of resources in the distinct practice locations by patients with NSBP and their assumed rationale for presenting to the ED. A diverse array of medical professionals was engaged through purposive sampling, leading to a non-probabilistic distribution of participants.

### Differences in care and resource utilization

Different diagnostic methodologies could be detected between EPs and GPs and within the group of emergency care providers.

Two primary strategies could be identified among EPs: a guideline-driven approach, focusing on basic physical examinations and prompt discharge, and a strategy that was driven by the availability of plentiful resources in the ED, involving extensive diagnostics (i.e. blood tests, imaging, and consultation of additional specialists).

The guideline-driven approach may represent a strategy aiming to quickly identify the potential for severe underlying conditions by a thorough history taking and physical exam. This approach ensured an efficient allocation of resources and is in line with clinical guidelines [29, 30]. Conversely, the abundance-driven approach reflected divergence from the guidelines and an alleged sense of diagnostic safety. Given the high prevalence of back pain presentation in EDs [31], a potential reason is the assumption of a higher incidence of serious conditions among the patients presenting in the ED with back pain, and the hence perceived justification for extensive diagnostics to rule out such conditions. However, this strategy resulted in greater resource consumption and could potentially impact healthcare quality negatively [32, 33]. Prior research indicates that the individual clinical decision-making is influenced by provider resources (i.e. risk tolerance, knowledge, work experience) and system resources (workload, costs/resources and distractions) [34, 35]. Hence, the finding of two oppositional approaches might indicate the need for educational measures among the physician staff to foster guideline adherence, especially in earlier career stages and overcrowding situations and in high turnover EDs.

GPs mostly pursued a gradual diagnostic approach, similar to the former strategy identified in the EP group. This approach likely benefits from the GPs’ capability to longitudinally observe patients and the advantage of ongoing doctor-patient relationships, enabling a continuous evaluation of the clinical course over time. Thereby, GPs are optimizing patient care within a framework that encourages judicious resource use and adherence to acknowledged medical guidelines [29, 30].

Regardless of the diagnostic approach pursued, the immediate therapeutic interventions were very similar across both groups of doctors and included the administration of pain medication and counselling, which is in line with clinical guidelines. Although the German guidelines for NSBP caution against the unreflected use of pain medication and highlight the need for education and reassurance of the benign prognosis [29], it is adequate to support timely symptom relief through pain medication. However, our interviews did not explore whether the pain medication was administered with the clear instruction of only being a short-term relief. Although the use of opioids for the treatment of acute and non-malignant back pain is not common in Germany [29, 36], our interview did not explore whether the pain medication administered was peripheral analgesics (e.g. acetaminophen, metamizole) or opioids. Additionally, counselling and psychosocial interventions also served the need to acknowledge and address patient concerns about a suspected serious cause of their pain. After the initial therapeutic measures above, the main recommendation for further care from both groups was a follow-up visit with the GP.

Regarding the recommendations for further treatment, the EPs seemed to focus on limiting resource expenditure, e.g. by advising to seek diagnostic imaging only if no improvement of symptoms was achieved. Also, a follow-up visit with their respective GP or over-the-counter pain medication was advised. Likewise, GPs aimed to establish a care pathway in the primary care setting and refrained from referring their patients to an ED. GPs mainly recommended physical therapy, but were also aware of specific movement programs that some statutory health insurances offered. Additionally, they focused on providing guidance for lifestyle modification (e.g. advice on ergonomic movement, establishing an active lifestyle, ongoing counselling, heat application, etc.) or specialist referrals. This indicated that both EPs and GPs deem the primary care sector as the better environment to treat patients presenting with NSBP sustainably. It furthermore highlighted that EPs predominantly focus on short-term symptom management without a focus on coordinating the primary care pathway beyond the referral to the GP. To us, this reflects that patients presenting to the ED with NSBP are misrouted in the healthcare system, partly due to the structure of the healthcare infrastructure and due to the different perception of urgency [33], leading to unnecessary fragmentation of their care and dissatisfaction for the healthcare providers involved in coordinating their respective care pathways.

### Providers perceptions of reasons for ED presentation

In our study, we recorded reasons for ED presentation reported by EPs or GPs. These reasons suggested a great variety and complex interaction of factors influencing patients to present to the ED and existing barriers to primary health care. The reported lack of sufficient knowledge about ambulant emergency care structures and the healthcare system in general indicates a possible target for further patient education and improving health literacy. These measures could be crucial to help patients navigate the healthcare system. Patients reportedly considered the ED a convenient option for receiving care at any time and the possibility of receiving comprehensive diagnostics and treatment in hours. These discrepant perceptions of the need for care in an ED are in line with prior research on this topic [37, 38]. Convenience has been considered an important factor for patients to seek medical care in EDs [39]. However, the all too convenient availability of the presentation in the ED seemed to drive over-utilization of resources and, potentially, the diversion of resources from patients requiring immediate care. Additionally, the dissatisfaction with ambulant care providers was reported by both groups as a driver, which highlights possible structural deficits in the ambulant care sector in Germany. Providers assumed that long waiting times for specialist consultation, diagnostic imaging and even GP appointments drive patients to EDs. As one interviewee pointed out, an increasing shortage of doctors in certain German areas could further aggravate the problem, which is in line with prior research [40]. These findings should be investigated in future studies to improve ambulatory care in general and primary care services in particular. Our results indicated that primary factors influencing ED diversion of patients with NSBP include convenience, fear or concern, heightened demand for diagnostic services, and insufficient knowledge regarding the organization of emergency care and are consistent with previous observations [24, 25].

### Strengths and limitations

In research methodologies, qualitative research designs focus on exploring patterns of behavior and perceptions rather than quantifying their prevalence within the population [41]. The identification of these patterns at the intersection between emergency medicine and primary care is an important strength of this research and allows addressing treatment, referral, and follow-up guidance for patients with back pain presenting in an ED. Yet, important limitations apply. One notable bias among interviewees is selection bias, as individuals who willingly participate may not represent the broader population. Selection bias may also result from the recruitment of interviewees from within the catchment and referral area of the Medical Center of the University of Freiburg. Additionally, the social desirability bias warrants consideration. When queried about their personal diagnostic and therapeutic approaches, interviewees may adjust their responses to perceived expectations, potentially compromising authenticity. Furthermore, the possibility of response or recall bias among interviewees must be acknowledged. We opted for video call interviews to enhance flexibility in scheduling, improve time efficiency, and facilitate easy recording. Granting interviewees the autonomy to select their interview setting, such as their practice or home environment, may foster a sense of comfort and encourage honest responses. However, it is important to note the potential for heightened interviewer bias inherent in video communication. Efforts were made to reduce biases by adhering to the structured interview format and predefined questions. Lastly, the case vignette presented to interviewees contained fewer detail than in other research [42], introducing the risk of diagnostic ambiguity. Yet, since the diagnosis of NSBP was explicitly mentioned in the introduction of the interview, we deem the risk for such ambiguity limited.

## Conclusions

We conducted a semi-structured interview study with EPs and GPs to assess the care and resource utilization in the respective care environment and the assumed reasons for ED presentation for patients with NSBP. This research identified three key insights:

Firstly, both groups agreed that the primary care sector can provide appropriate care for patients with NSBP. Secondly, diagnostic approaches differ. In the ED, we identified two different approaches. The first approach showed high compliance with clinical guidelines, focusing on clinical reasoning and ruling out red flags. In this approach, therapeutic means were limited to symptom control and the referral to further management in the primary care sector. This was similar to the approach pursued by most of the GPs interviewed. However, a second approach in the ED was identified, which resulted in high resource consumption from broad diagnostic measures. Junior doctors were found to be more prone to the latter approach, which could reflect insecurity and imply a potential need for training. Furthermore, this might be affected by the expectation expressed by some patients to receive a thorough diagnostic workup when presenting to the ED. Likewise, this implies the need for further training. Thirdly, the perceived reasons for ED presentation of patients with NSBP. These included insufficient knowledge of healthcare infrastructure, their wish for a fast resolution of symptoms, the convenience of presenting to the ED, as well as the patients’ concerns and increased demand for diagnostics. Future research should investigate strategies to increase adherence to guidelines to reduce overutilization of resources for patients presenting with NSBP.

## Electronic supplementary material

Below is the link to the electronic supplementary material.

Supplementary Material 1

Supplementary Material 2

## Data Availability

The interviews were conducted after consent was obtained. The recorded interviews, the transcripts and the software analyses performed are locally stored and can be made available upon reasonable request.

## Abbreviations

ED: Emergency Department
EP: Emergency Physician
GP: General Practitioner
NSBP: Non-specific Back Pain
