# Supplementary material for: Provider perception of presentations with nonspecific back pain in the emergency department and primary care practices: a semi-structured interview study
Source: Int J Emerg Med. 2024 Sep 11;17:121. doi: 10.1186/s12245-024-00694-2 (PMC11389560; doi:10.1186/s12245-024-00694-2)
Supplement: Supplementary file 1 — Supplementary Material 1 [file 12245_2024_694_MOESM1_ESM.docx]

# Supplement

Supplementary Table 1. Case presentation and sequence of consecutive interview questions

| Case | Imagine a patient with non-specific back pain. There is no prior history of trauma, infection, or inflammation. There is no past medical history regarding malignancies. |
| --- | --- |
|  | Optional clarifying question by interviewee. |
| Question 1 | What would your typical diagnostic and therapeutic approach to such a patient look like? |
| Question 2 | In your opinion, what are the reasons why patients present to an emergency department with low-acuity and non-specific back pain described above? |
| Question 3 | In your opinion, what are the differences in care of these patients in an emergency department or ambulant care practice? |
| Question 4 | Following the acute presentation, you advise the patient on how to proceed. What advice would you usually give such a patient? |
| Question 5 | In your opinion, have we forgotten any important aspects for this patient group? |
