# Supplementary material for: Provider perception of presentations with nonspecific back pain in the emergency department and primary care practices: a semi-structured interview study
Source: Int J Emerg Med. 2024 Sep 11;17:121. doi: 10.1186/s12245-024-00694-2 (PMC11389560; doi:10.1186/s12245-024-00694-2)
Supplement: Supplementary file 2 — Supplementary Material 2 [file 12245_2024_694_MOESM2_ESM.docx]

**Supplementary Table 1:** COREQ Checklist.

| **Nr.** | **Item** | **Implementation** |
| --- | --- | --- |
|  | **Domain 1: Research team and reflexivity** |  |
|  | *Personal characteristics* |  |
| 1 | Interviewer/facilitator | Interviews were conducted by NK |
| 2 | Researchers credentials | M.D. |
| 3 | Occupation | Resident physician |
| 4 | Gender | Female |
| 5 | Experience/training | 3 years work experience as a resident physician in internal medicine |
|  | *Relationship with participants* |  |
| 6 | Relationship established | No prior relationship was established for 14 of 16 participants. Two participants were remotely acquainted with the interviewer (one former colleague, one relative of a colleague). |
| 7 | Participant knowledge of the interviewer | Reasons for study was communicated, interviewer was introduced as a doctoral researcher at the Medical Faculty of the University of Freiburg. |
| 8 | Interviewer characteristics | Upon request, the interviewer disclosed to be a resident physician in the field of internal medicine / emergency medicine and a doctoral researcher at the Medical Faculty of the University of Freiburg. |
|  | **Domain 2: study design** |  |
|  | *Theoretical framework* |  |
| 9 | Methodological orientation and Theory | The methodology of qualitative content analysis was used, based on Mayring [^3^](https://paperpile.com/c/f2yRmc/RfIE) and MacQueen et al. [^4^](https://paperpile.com/c/f2yRmc/OrQJ) |
|  | *Participant selection* |  |
| 10 | Sampling | Purposive sampling of EPs and GPs |
| 11 | Method of approach | Participants were approached by mail (center-wide newsletter) |
| 12 | Sample size | 16 interviewees participated in this study. Of those, seven were EPs and nine were GPs. |
| 13 | Non-participation | Two persons dropped out after initial approval. Reasons for dropping out were not given to the authors. |
|  | *Setting* |  |
| 14 | Setting of data collection | The interviews were conducted via the software *zoom.* Details are provided in the Methods section of the manuscript. |
| 15 | Presence of non-participants | No one other than the interviewer and interviewee were present in the interview. |
| 16 | Description of sample | see Table 1 in manuscript. |
|  | *Data collection* |  |
| 17 | Interview guide | Questions were generally not shared with the interviewees before the interview. One participant wished to know the questions beforehand, which was granted. The interview was pilot tested on two physicians from the same source population of EPs and GPs. |
| 18 | Repeat interviews | Every interview was conducted once. |
| 19 | Audio/visual recording | Interviews were audio recorded. |
| 20 | Field notes | No field notes were taken during or after the interviews. |
| 21 | Duration | see Table 1 in manuscript. |
| 22 | Data saturation | Data saturation was declared as needed and assessed following the publication of Guest et al.[^5^](https://paperpile.com/c/f2yRmc/aVIC). |
| 23 | Transcripts returned | Transcripts were proof red by NK and not returned to the participants for comments. |
|  | **Domain 3: Analysis and findings** |  |
|  | *Data analysis* |  |
| 24 | Number of data coders | Codes were generated by NK and LB. |
| 25 | Description of the coding tree | The coding tree was structured to reflect the topics of the questions s. Additional new aspects were integrated through an incremental, iterative process. |
| 26 | Derivation of themes | Major themes were set in advance, minor themes were identified during the interviews. |
| 27 | Software | The software MaxQDA 2022 was used. Details are provided in the Methods section of the manuscript. |
| 28 | Participant checking | No feedback from participants regarding the findings was provided. |
|  | *Reporting* |  |
| 29 | Quotations presented | Quotations were presented to illustrate findings and marked with the participant number. |
| 30 | Data and findings consistent | Data and findings were consistent. |
| 31 | Clarity of major themes | Major themes were presented in the findings by structuring them in sections. |
| 32 | Clarity of minor themes | Not all minor themes were not all included in the article to ensure legibility of the manuscript. |

1. [Jo, S. *et al.* Emergency department occupancy ratio is associated with increased early mortality. *J. Emerg. Med.* 46, 241–249 (2014).](http://paperpile.com/b/f2yRmc/fA5RK)

2. [Sun, B. C. *et al.* Effect of emergency department crowding on outcomes of admitted patients. *Ann. Emerg. Med.* 61, 605–611.e6 (2013).](http://paperpile.com/b/f2yRmc/65sg9)

3. [Mayring, P. *Qualitative Inhaltsanalyse: Grundlagen und Techniken*. (Beltz, 2015).](http://paperpile.com/b/f2yRmc/RfIE)

4. [MacQueen, K. M., McLellan, E., Kay, K. & Milstein, B. Codebook Development for Team-Based Qualitative Analysis. *CAMSI J.* 10, 31–36 (1998).](http://paperpile.com/b/f2yRmc/OrQJ)

5. [Guest, G., Bunce, A. & Johnson, L. How Many Interviews Are Enough?: An Experiment with Data Saturation and Variability. *Field methods* 18, 59–82 (2006).](http://paperpile.com/b/f2yRmc/aVIC)
